# Cigarette Smoking and the Risk of Bladder Cancer in Men and Women

**DOI:** 10.1186/1617-9625-2-3-141

**Published:** 2004-09-15

**Authors:** Jeffrey T Quirk, Qiang Li, Nachimuthu Natarajan, Curtis J Mettlin, K Michael Cummings

**Affiliations:** 1 Department of Biology and Health Services, Edinboro University of Pennsylvania, Edinboro, Pennsylvania, USA; 2 Department of Health Behavior, Roswell Park Cancer Institute, Buffalo, New York, USA; 3 Division of Cancer Prevention and Population Sciences, Roswell Park Cancer Institute, Buffalo, New York, USA

## Abstract

Although cigarette smoking is a principal risk factor for bladder cancer in both men and women, few studies have statistically evaluated whether gender modifies the effect of smoking on bladder cancer risk. We initiated the present case-control study at Roswell Park Cancer Institute in Buffalo, New York, U.S., to provide further data on this important issue. We observed similar risk estimates for men and women with comparable smoking exposures, but did not observe a statistically significant interaction between gender and lifetime smoking exposure. We conclude that cigarette smoking is a major risk factor for bladder cancer in both sexes, but that gender does not modify the effect of smoking on bladder cancer risk.

## Introduction

Cigarette smoking is a major independent risk factor for urinary bladder cancer. Many epidemiologic studies conducted in various parts of the world have shown that both male and female cigarette smokers have two to four times the risk of developing bladder cancer compared to nonsmokers. In general, the risk increases as the frequency and duration of smoking levels increase, and risk typically decreases in former smokers compared to current smokers [1,2].

In a recent case-control study conducted in Los Angeles, California, U.S., Castelao and colleagues [3] reported that when men and women smoked comparable amounts of cigarettes, women incurred a higher risk of bladder cancer than men. This conclusion was based in part from observing a statistically significant interaction between sex and lifetime cigarette consumption in a regression model that predicted the risk of bladder cancer. This finding, if confirmed, would have implications for our understanding of the role of gender in the etiology of bladder cancer. In an effort to provide more data on this important issue, we conducted a hospital-based case-control study to investigate the relationship between cigarette smoking and bladder cancer, and statistically evaluated whether gender was a potential effect modifier for the association between smoking and the risk of bladder cancer.

## Subjects and Methods

This study was approved by the Institutional Review Board of Roswell Park Cancer Institute (RPCI) in Buffalo, New York, U.S., and informed consent was properly obtained from all participants. Study subjects included adult patients admitted to RPCI between 1982 and 1998, who volunteered to complete a self-administered epidemiologic questionnaire. The details of this hospital-based data collection system have been previously described [4]. Cases (n = 499; 381 males and 118 females) included patients diagnosed with primary, histologically confirmed bladder cancer. Over 93% (466/499) of the malignant neoplasms were classified as transitional cell carcinomas, which account for roughly 90% of bladder cancer presentations in the United States [5]. Cases were predominantly white (97%) and ranged in age from 24 to 90 years (mean age = 65.3 ± 10.7 years). Seventy-five percent (375/499) of cases were 60 years of age and older. Controls (n = 1,922; 1,447 males and 475 females) included patients seen at RPCI who were discharged without a diagnosis of malignancy. Among a pool of 8,733 eligible patients, controls were frequency-matched to cases by sex and five-year age intervals for a final case-to-control ratio of approximately 1:4. Similar to cases, controls were predominantly white (99%) and ranged in age from 19 to 94 years (mean age = 64.8 ± 10.9 years). The most frequently used clinical services for the male control patients included urology (23%), surgery (15%), and dermatology (12%), and the most common diagnoses included diseases of the digestive system (27%), diseases of the circulatory system (24%), diseases of the breast and genito-urinary systems (11%), and diseases of the respiratory system (8%). The most frequently used clinical services for the female control patients included breast (22%), gynecology (17%), and surgery (14%), and the most common diagnoses included diseases of the breast and genito-urinary systems (30%), diseases of the circulatory system (26%), and diseases of the digestive system (25%).

A patient was classified as an ever smoker, or never smoker, if he/she reported having smoked, or having not smoked, cigarettes every day for at least one year during his/her lifetime. Ever smokers were further classified as current or former smokers depending on whether the patient had reported quitting smoking at the time of questionnaire completion. Smoking histories were measured by the total number of years the patient reported smoking (duration), and the usual number of cigarettes smoked daily during years of regular use (frequency). A summary measure of lifetime smoking exposure (pack-years) was calculated as the product of frequency (packs/day) × duration (years).

Unconditional multiple logistic regression was used to calculate odds ratio (OR) and 95% confidence interval (CI) estimates, and to test for the statistical significance of risk trends. OR and 95% CI estimates presented in this report are adjusted solely for age, which was entered as a continuous variable. Further adjustment for potential confounders such as occupational exposures (gasoline/oil, textiles, dyes, and chemicals/acids/solvents), education, income, and geographic area of residence failed to notably alter point estimates. Gender was evaluated as a potential effect modifier for the association between smoking and the risk of bladder cancer by testing for the statistical significance of an interaction term (i.e., the product of pack-years of smoking and sex) in a logistic regression model [6]. All data analyses were performed using SPSS for Windows version 10.0 (SPSS Inc., Chicago, Illinois). Statistical tests were two-sided, and the level of significance was set at α = 0.05.

## Results and Discussion

Subject cigarette smoking histories and the results from the logistic regression analyses are presented in Table 1. Approximately 80% percent of cases and 63% of controls were classified as ever smokers. Among ever smokers, 31% of cases and 23% of controls were current smokers. Compared to never smokers, ever smokers had a statistically significant two-fold increased risk for bladder cancer in the total subject population (OR = 2.4; 95% CI = 1.9 to 3.0), in males (OR = 2.6; 95% CI = 1.9 to 3.5), and in females (OR = 2.1; 95% CI = 1.4 to 3.2). Male current smokers (OR = 4.2; 95% CI = 2.9 to 6.1) had a markedly increased risk for bladder cancer compared to female current smokers (OR = 2.1; 95% CI = 1.2 to 3.7). Compared to current smokers, former smokers had reduced risks of bladder cancer in the total subject population and in males, but not in females.

**Table 1:** Subject cigarette smoking histories and the risk of bladder cancer, Roswell Park Cancer Institute, 1982–1998^*a*^

	**Total Subject Population**			**Males**			**Females**		
	**Cases**	**Controls**	**OR (95% CI)^*b*^**	**Cases**	**Controls**	**OR (95% CI)**	**Cases**	**Controls**	**OR (95% CI)**
Never smokers	100	718	1.0 (referent)	60	472	1.0 (referent)	40	246	1.0 (referent)
Ever smokers	396	1198	2.4 (1.9–3.0)	318	970	2.6 (1.9–3.5)	78	228	2.1 (1.4–3.2)
(Missing)	(3)	(6)		(3)	(5)			(1)	
Current smokers	122	271	3.4 (2.5–4.6)	95	190	4.2 (2.9–6.1)	27	81	2.1 (1.2–3.7)
Former smokers	274	927	2.1 (1.6–2.7)	223	780	2.2 (1.6–3.0)	51	147	2.1 (1.3–3.4)
**Pack-years of smoking**									
≤ 10	27	192	1.0 (0.64–1.6)	18	131	1.1 (0.62–1.9)	9	61	0.91 (0.42–2.0)
11–20	44	182	1.7 (1.2–2.6)	32	145	1.7 (1.1–2.8)	12	37	2.0 (0.94–4.2)
21–30	41	172	1.7 (1.2–2.6)	33	143	1.8 (1.1–2.9)	8	29	1.7 (0.72–4.0)
31–40	52	166	2.3 (1.5–3.3)	40	136	2.3 (1.5–3.6)	12	30	2.5 (1.2–5.2)
> 40	218	436	3.6 (2.8–4.7)	185	374	3.9 (2.8–5.4)	33	62	3.3 (1.9–5.6)
(Missing)	(14)	(50)		(10	(41)		(4)	(9)	
*P *for trend^*c*^			< .001			< .001			.004
**Duration (years of smoking)**									
1–10	19	134	1.0 (0.60–1.7)	14	103	1.1 (0.57–2.0)	5	31	0.98 (0.36–2.7)
11–20	47	215	1.6 (1.1–2.3)	38	181	1.6 (1.1–2.6)	9	34	1.6 (0.70–3.6)
21–30	69	250	2.0 (1.4–2.8)	52	200	2.0 (1.4–3.1)	17	50	2.1 (1.1–4.0)
31–40	101	275	2.6 (1.9–3.6)	82	223	2.9 (2.0–4.2)	19	52	2.2 (1.2–4.2)
> 40	153	301	3.7 (2.8–4.9)	127	244	4.1 (2.9–5.8)	26	57	2.8 (1.6–5.0)
(Missing)	(7)	(23)		(5)	(19)		(2)	(4)	
*P *for trend			< .001			< .001			.022
**Frequency (cigarettes per day)**									
1–10	45	230	1.4 (0.96–2.1)	30	149	1.6 (0.99–2.6)	15	81	1.1 (0.60 – 2.2)
11–20	168	506	2.4 (1.8–3.1)	131	415	2.5 (1.8–3.4)	37	91	2.5 (1.5 – 4.2)
21–30	51	170	2.2 (1.5–3.2)	42	141	2.4 (1.5–3.7)	9	29	1.9 (0.84 – 4.4)
31–40	90	187	3.5 (2.5–4.9)	80	168	3.8 (2.6–5.6)	10	19	3.3 (1.4 – 7.6)
> 40	33	79	3.0 (1.9–4.8)	29	74	3.1 (1.9–5.2)	4	5	4.9 (1.3 – 19.2)
(Missing)	(9)	(26)		(6)	(23)		(3)	(3)	
*P *for trend			< .001			< .001			.005

^*a*^Adjusted for age

^*b*^OR = odds ratio, CI = confidence interval

^*c*^P = probability

The risk of bladder cancer generally increased in both sexes as pack-years, duration, and frequency levels increased. We observed similar risk estimates for males and females with comparable smoking histories, and did not find a statistically significant interaction between lifetime smoking exposure and sex (P = 0.76), thereby leading us to conclude that gender was not an effect modifier for the association between smoking and bladder cancer in our case-control data. In the two other case-control studies that have formally tested for the statistical significance of an interaction term (i.e., the product of lifetime cigarette consumption and sex), Castelao and colleagues [3] detected a statistically significant interaction (P = .016), while Burch and colleagues [7] did not (P = 0.13).

The results of this study are consistent with those of other recent case-control studies from the United States [8-10] and Canada [7], which have reported approximately equivalent smoking-related risk estimates for bladder cancer in males and females. Recent combined analyses of eleven case-control studies from Europe [11,12] also found that gender did not modify the association between smoking and bladder cancer, with males and females sharing nearly identical risks regardless of duration or frequency levels.

It is important to consider that in most populations worldwide, bladder cancer is relatively rare in women. Men have approximately three to four times the risk of developing bladder cancer compared to women, and this sex difference in risk persists even after controlling for differences in smoking and occupational exposures [1,2,5,9]. The exact causes for the observed excess risk of bladder cancer in men are currently not known. Future research should continue to explore possible explanations for this excess risk, including potential sex differences in genetic, anatomic, and hormonal factors, urination habits, and perhaps previously unidentified environmental risk factors [1,9,13]. In summary, in this case-control investigation we found that cigarette smoking was an important risk factor for bladder cancer in both sexes, and that for comparable cigarette smoking exposures, men and women shared similar risk estimates for bladder cancer.

## Competing interests

The authors declare that they have no competing interests.
